# Body Adiposity Index versus Body Mass Index and Other Anthropometric Traits as Correlates of Cardiometabolic Risk Factors

**DOI:** 10.1371/journal.pone.0065954

**Published:** 2013-06-11

**Authors:** Charlene T. Lichtash, Jinrui Cui, Xiuqing Guo, Yii-Der I. Chen, Willa A. Hsueh, Jerome I. Rotter, Mark O. Goodarzi

**Affiliations:** 1 Department of Medicine, Cedars-Sinai Medical Center, Los Angeles, California, United States of America; 2 Medical Genetics Institute, Cedars-Sinai Medical Center, Los Angeles, California, United States of America; 3 Diabetes Research Center, Division of Diabetes, Obesity and Lipids, Methodist Hospital Research Institute, Houston, Texas, United States of America; 4 Division of Endocrinology, Diabetes and Metabolism, Cedars-Sinai Medical Center, Los Angeles, California, United States of America; 5 Department of Medicine, David Geffen School of Medicine, University of California Los Angeles, Los Angeles, California, United States of America; University of Tor Vergata, Italy

## Abstract

**Objective:**

The worldwide prevalence of obesity mandates a widely accessible tool to categorize adiposity that can best predict associated health risks. The body adiposity index (BAI) was designed as a single equation to predict body adiposity in pooled analysis of both genders. We compared body adiposity index (BAI), body mass index (BMI), and other anthropometric measures, including percent body fat (PBF), in their correlations with cardiometabolic risk factors. We also compared BAI with BMI to determine which index is a better predictor of PBF.

**Methods:**

The cohort consisted of 698 Mexican Americans. We calculated correlations of BAI, BMI, and other anthropometric measurements (PBF measured by dual energy X-ray absorptiometry, waist and hip circumference, height, weight) with glucose homeostasis indices (including insulin sensitivity and insulin clearance from euglycemic clamp), lipid parameters, cardiovascular traits (including carotid intima-media thickness), and biomarkers (C-reactive protein, plasminogen activator inhibitor-1 and adiponectin). Correlations between each anthropometric measure and cardiometabolic trait were compared in both sex-pooled and sex-stratified groups.

**Results:**

BMI was associated with all but two measured traits (carotid intima-media thickness and fasting glucose in men), while BAI lacked association with several variables. BAI did not outperform BMI in its associations with any cardiometabolic trait. BAI was correlated more strongly than BMI with PBF in sex-pooled analyses (r = 0.78 versus r = 0.51), but not in sex-stratified analyses (men, r = 0.63 versus r = 0.79; women, r = 0.69 versus r = 0.77). Additionally, PBF showed fewer correlations with cardiometabolic risk factors than BMI. Weight was more strongly correlated than hip with many of the cardiometabolic risk factors examined.

**Conclusions:**

BAI is inferior to the widely used BMI as a correlate of the cardiometabolic risk factors studied. Additionally, BMI’s relationship with total adiposity may not be the sole determinate of its association with cardiometabolic risk.

## Introduction

The prevalence of obesity worldwide and across age groups has made it a focus of much investigative research. In the United States, the prevalence of obesity has been estimated at approximately one-third of the population, with the combined proportion of overweight and obese individuals encompassing approximately two thirds of the country [1]. Obesity is associated with an increased incidence of poor health outcomes, including cardiovascular disease, hypertension, diabetes mellitus, dyslipidemia, osteoarthritis, and certain cancers [2].

The most widely used method to categorize overweight and obese individuals is the body mass index (BMI, (weight in kilograms)/(height in meters)^2^), first named the Quetelet index and described by Adolphe Quetelet in 1832 [3]. In 1998, the National Heart, Lung, and Blood Institute of the National Institutes of Health (NIH) published clinical guidelines in which BMI was used in the systematic classification of overweight and obese individuals [4].

However, BMI is an imperfect measure of body adiposity. The weight term in BMI does not distinguish between muscle mass and fat mass. Furthermore, BMI has been shown to be age-, sex-, and in some cases, race-, dependent [5], [6]. At equivalent BMIs, women have significantly greater amounts of total body fat than men, and older individuals have significantly greater amounts of total body fat than those who are younger [5], [6]. Finally, it does not describe the depot of total fat in a person. Despite these disparities, however, the same BMI cutpoints for overweight and obesity are often applied across sex and age groups.

In May 2011, Bergman *et al*. introduced a new parameter, the body adiposity index (BAI, (hip circumference in centimeters)/(height in meters)^1.5^–18), derived via analyses of Mexican-American subjects, and validated in African-Americans [7]. Among its proposed advantages, the BAI presents a method of estimating body adiposity without requiring assessment of body weight, offering a simple-to-use tool that can be accessed globally [7]. Additionally, the demonstration of similar linear relationships between BAI and percent body fat (PBF) in men and women suggests that sex-specific adjustments of BAI as an estimate of PBF may not be necessary [7].

Several subsequent studies evaluated BAI as a predictor of body fat composition, yielding inconsistent results. While BAI was more strongly correlated than BMI with dual-energy X-ray absorptiometry (DXA)-derived PBF in sex-pooled analyses [8], [9], results have varied in sex-stratified analyses, with BAI being similarly correlated [8], [10] or less correlated [9] than BMI with PBF. Conflicting results have also been observed in states of extreme adiposity; BAI was more correlated than BMI with PBF in women with familial partial lipodystrophy [11]. In contrast, in a study of severely obese women, BMI, but not BAI, was significantly correlated with DXA-derived PBF [12].

To date, several published studies have examined BAI’s association with health risks or outcomes [9], [13], [14], [15], [16], [17], [18]. Findings of the studies reported thus far illustrate the need for further clarification on the clinical utility of BAI as a measure of body adiposity and correlate of disease [13], [19]. The goal of our study was to define the correlations of BAI, BMI, and other measured anthropometric variables with (a) glucose homeostasis traits, (b) lipid parameters, (c) cardiovascular traits, and (d) biomarkers. In cases wherein both BMI and BAI were associated with a trait, we statistically assessed whether one was more strongly associated than the other, to elucidate which may be more associated with cardiometabolic risk. A secondary aim of our study was to examine the associations of the various anthropometric variables with PBF, and to determine the correlation of PBF itself with cardiometabolic risk factors.

## Materials and Methods

### Study Subjects

Metabolic and cardiovascular phenotypes were assessed in participants of the UCLA/Cedars-Sinai Mexican-American Coronary Artery Disease (MACAD) project, a study of Mexican-American families from Los Angeles [20], [21]. To be classified as Mexican and qualify for the study, subjects had to report at least three grandparents of Mexican origin. In the present report, 698 subjects from 193 families (299 male and 399 female) with BAI values were studied, comprising adult offspring (age 18 or older) of probands with coronary artery disease, and the spouses of those offspring (if available) [20], [21]. By design, offspring were free of overt cardiovascular and metabolic disease, thus avoiding secondary changes in phenotype caused by overt disease.

### Ethics Statement

All studies were approved by Human Subjects Protection Institutional Review Boards at UCLA and Cedars-Sinai Medical Center. All subjects gave written informed consent prior to participation.

### Phenotyping Procedures

Subjects underwent a phenotyping protocol that included testing of glucose homeostasis indices, lipid parameters, cardiovascular traits, and biomarkers. In the original MACAD study design, a three-day phenotyping protocol was to take place within one week for each subject in the offspring generation. As executed, the time window for completion of all phenotyping ranged from one week to several years. The median time to completion of studies was 25.5 days; 81% of subjects completed phenotyping within 6 months. Only subjects completing studies within one year were included in the current study. On one day, fasting blood was obtained, followed by a 75 g oral glucose tolerance test (OGTT). On a separate day, B-mode ultrasound was performed for measurement of common carotid artery intima-media thickness (IMT) and dual-energy X-ray absorptiometry (DXA) scan was performed to assess body fat distribution. On a further day, a euglycemic-hyperinsulinemic clamp was performed.

During the clamp, a priming dose of human insulin (Novolin, Clayton, NC) was given and followed by infusion for 120 minutes at a constant rate (60 mU·m^−2^·min^−1^) with the goal of achieving a steady state plasma insulin concentration of 100 µIU/ml or greater [20], [22]. Blood was sampled every 5 minutes, and the rate of 20% dextrose coinfused was adjusted to maintain plasma glucose concentrations at 95 to 100 mg/dl. The glucose infusion rate (M value, mg·m^−2^·min^−1^) over the last 30 minutes of steady-state insulin and glucose concentrations reflects glucose uptake by all tissues of the body (primarily insulin-mediated glucose uptake in muscle) and is therefore directly correlated with tissue insulin sensitivity [22]. The insulin sensitivity index (M/I, mg·m^−2^·min^−1^·µIU^−1^·mL) was calculated as M divided by the steady state plasma insulin level (I). The metabolic clearance rate of insulin (MCRI, mL·m^−2^·min^−1^) was calculated as the insulin infusion rate divided by the steady state insulin level of the euglycemic clamp, as previously described [22], [23].

Fasting lipid parameters including low-density lipoprotein cholesterol (LDL-C), high-density lipoprotein cholesterol (HDL-C), and triglycerides (TG), were examined in this study.

Cardiovascular traits included systolic and diastolic blood pressure (SBP and DBP) and carotid intima-media thickness (IMT) [24], [25]. Carotid artery images were obtained by high-resolution B-mode ultrasound using the Toshiba SSH-140A ultrasound system with a 7.5-MHz probe, at the University of Southern California Atherosclerosis Research Unit [26]. The IMT measure represents the distance between the blood-intima and media-adventitia echoes taken at the right distal common carotid artery [26]. Values are reported as the average of 80 to 100 individual IMT measurements made over 1 cm of the right distal common carotid artery [26].

Fasting biomarkers included C-reactive protein (CRP), adiponectin, and plasminogen activator inhibitor-1 (PAI-1) levels.

Also tested were percent body fat (PBF) measured by DXA, as well as anthropometric indices (BMI, BAI, height, waist circumference, weight, and hip circumference) [27].

### Data Analysis

Log-transformed (BAI, BMI, weight, hip, HDL-C, TG, carotid IMT, adiponectin, CRP, 2-hour glucose, fasting insulin) or square-root transformed (M/I, MCRI, PAI-I) trait values were used to normalize the distribution for statistical analyses. We computed correlation coefficients (r) between the cardiometabolic phenotypes mentioned above, and the following measures: BMI, BAI, waist, hip, height, weight, and PBF and the *P* values indicating the difference of the correlation coefficients from zero. Correlation coefficients were also used to compare anthropometric measures in their degree of association with PBF. Hotelling’s t-test was used to determine whether correlation coefficients were significantly different. *P* values of <0.05 were considered significant.

Because the MACAD cohort consists of families, we also computed correlation coefficients using generalized estimating equations (GEE), adjusting for familial relationships. The weighted GEE1 [28] was computed assuming an exchangeable correlation structure and using the sandwich estimator of the variance to account for familial correlation present in family data.

## Results

### General Characteristics

The clinical characteristics of the 698 subjects are shown in Table 1. BMI did not differ significantly by gender, while BAI was higher in women than in men. Of the anthropometric measurements, waist circumference, weight, and height were significantly higher in men, while hip circumference and PBF were higher in women. With the exception of MCRI, fasting insulin, and PAI-1, all of the cardiovascular and metabolic phenotypes differed significantly between men and women. Men had more adverse lipid and cardiovascular profiles, while no clear pattern was observed within the glucose homeostasis and biomarker categories.

**Table 1:** Clinical characteristics of the study cohort.

	Women (n = 399)	Men(n = 299)	*P* value
Age (yr)	34.0 (13.0)	34.0 (14.0)	0.998
BMI (kg/m^2^)	28.3 (7.0)	28.6 (5.7)	0.78
BAI (%)	35.4 (8.3)	28.5 (5.1)	<0.0001
Waist Circumference (cm)	89.0 (17.3)	96.5 (13.2)	<0.0001
Hip Circumference (cm)	104.5 (15.4)	103.3 (10.9)	0.0032
Weight (kg)	69.4 (17.9)	82.0 (17.7)	<0.0001
Height (cm)	157.0 (6.9)	170.0 (7.9)	<0.0001
PBF (%)	38.3 (7.7)	24.9 (6.3)	<0.0001
M/I (mg·m^−2^·min^−1^·µIU^−1^·mL)	1.7 (1.2)	1.9 (1.6)	0.0037
MCRI (mL·m^−2^·min^−1^)	468.1 (128.7)	470.0 (137.4)	0.75
Fasting glucose (mmol/L)	5.05 (0.72)	5.32 (0.64)	0.0028
2-hour glucose (mmol/L)	6.38 (2.41)	5.77 (2.64)	0.0001
Fasting insulin (pmol/L)	74.4 (51.0)	67.8 (46.8)	0.61
LDL-C (mmol/L)	2.70 (0.92)	2.91 (1.04)	0.0008
HDL-C (mmol/L)	1.22 (0.41)	1.06 (0.34)	<0.0001
TG (mmol/L)	1.12 (0.85)	1.50 (1.21)	<0.0001
Carotid IMT (mm)	0.63 (0.1)	0.66 (0.1)	0.0086
SBP (mmHg)	109.3 (16.3)	116.7 (17.3)	<0.0001
DBP (mmHg)	64.7 (12.0)	69.3 (10.8)	<0.0001
CRP (mg/L)	2.0 (2.4)	1.1 (1.2)	<0.0001
Adiponectin (µg/mL)	7.8 (4.6)	6.3 (3.3)	0.0002
PAI-1 (ng/mL)	33.2 (26.9)	34.6 (31.0)	0.29

Data are medians (interquartile range).

BAI, body adiposity index; BMI, body mass index; Carotid IMT, carotid intima-media thickness; CRP, C-reactive protein; DBP, diastolic blood pressure; HDL-C, high density lipoprotein cholesterol; LDL-C, low density lipoprotein cholesterol; MCRI, metabolic clearance rate of insulin; M/I, insulin sensitivity index from the euglycemic-hyperinsulinemic clamp; PAI-1, plasminogen activator inhibitor-1; PBF, percent total body fat; SBP, systolic blood pressure; TG, triglycerides.

### Correlations of Anthropometric Measurements with Cardiometabolic Risk Factors

We found that waist circumference, weight, and BMI were significantly correlated with all lipid parameters, glucose homeostasis traits, cardiovascular traits, and biomarkers, with the exception of carotid IMT, LDL-C, and fasting glucose in men (Table 2). Hip circumference was correlated with fewer of the cardiometabolic variables; lack of correlation was found with DBP and LDL-C in both sex-pooled and sex-stratified analyses, and with 2-hour glucose, fasting glucose, adiponectin levels, and carotid IMT in men. Comparatively, height showed the least number of significant relationships with the cardiometabolic variables. BAI and PBF shared a comparable pattern of lack of correlation, with neither having significant associations with LDL-C, TG, fasting glucose, carotid IMT, adiponectin, and SBP in sex-pooled analyses, as well as adiponectin in women and carotid IMT in men. We also conducted correlation analyses taking family relationships into account, and found that the correlation coefficients were essentially the same (Table S1).

**Table 2:** Correlations of anthropometric measurements with cardiometabolic risk factors.

		Waist	Hip	Height	Weight	BAI	BMI	PBF
PBF (DXA)	All	0.28 (*P*<0.0001)	0.53 (*P*<0.0001)	−0.54 (*P*<0.0001)	0.15 (*P* = 0.0001)	0.78 (*P*<0.0001)	0.51 (*P*<0.0001)	
	Men	0.77 (*P*<0.0001)	0.70 (*P*<0.0001)	0.087 (*P* = 0.15)	0.76 (*P*<0.0001)	0.63 (*P*<0.0001)	0.79 (*P*<0.0001)	
	Women	0.67 (*P*<0.0001)	0.68 (*P*<0.0001)	−0.041 (*P* = 0.44)	0.71 (*P*<0.0001)	0.69 (*P*<0.0001)	0.77 (*P*<0.0001)	
LDL-C	All	0.19 (*P*<0.0001)	0.076 (*P* = 0.056)	0.041 (*P* = 0.30)	0.15 (*P* = 0.0002)	0.033 (*P* = 0.40)	0.14 (*P* = 0.0003)	−0.008 (*P* = 0.85)
	Men	0.15 (*P* = 0.016)	0.11 (*P* = 0.088)	−0.062 (*P* = 0.32)	0.10 (*P* = 0.10)	0.15 (*P* = 0.015)	0.14 (*P* = 0.024)	0.072 (*P* = 0.27)
	Women	0.18 (*P* = 0.0004)	0.087 (*P* = 0.095)	−0.088 (*P* = 0.089)	0.11 (*P* = 0.031)	0.12 (*P* = 0.021)	0.16 (*P* = 0.0026)	0.18 (*P* = 0.0011)
HDL-C	All	−0.32 (*P*<0.0001)	−0.22 (*P*<0.0001)	−0.27 (*P*<0.0001)	−0.37 (*P*<0.0001)	−0.008 (*P* = 0.84)	−0.27 (*P*<0.0001)	0.11 (*P* = 0.0065)
	Men	−0.31 (*P*<0.0001)	−0.26 (*P*<0.0001)	−0.10 (*P* = 0.095)	−0.33 (*P*<0.0001)	−0.21 (*P* = 0.0005)	−0.32 (*P*<0.0001)	−0.26 (*P*<0.0001)
	Women	−0.26 (*P*<0.0001)	−0.26 (*P*<0.0001)	−0.11 (*P* = 0.040)	−0.29 (*P*<0.0001)	−0.21 (*P*<0.0001)	−0.27 (*P*<0.0001)	−0.13 (*P* = 0.022)
TG	All	0.36 (*P*<0.0001)	0.18 (*P*<0.0001)	0.15 (*P* = 0.0001)	0.36 (*P*<0.0001)	0.054 (*P* = 0.17)	0.32 (*P*<0.0001)	−0.031 (*P* = 0.45)
	Men	0.35 (*P*<0.0001)	0.23 (*P* = 0.0001)	0.013 (*P* = 0.83)	0.33 (*P*<0.0001)	0.23 (*P*<0.0001)	0.36 (*P*<0.0001)	0.26 (*P*<0.0001)
	Women	0.31 (*P*<0.0001)	0.21 (*P*<0.0001)	−0.078 (*P* = 0.13)	0.27 (*P*<0.0001)	0.24 (*P*<0.0001)	0.33 (*P*<0.0001)	0.21 (*P*<0.0001)
M/I	All	−0.42 (*P*<0.0001)	−0.41 (*P*<0.0001)	0.045 (*P* = 0.27)	−0.37 (*P*<0.0001)	−0.37 (*P*<0.0001)	−0.46 (*P*<0.0001)	−0.36 (*P*<0.0001)
	Men	−0.53 (*P*<0.0001)	−0.47 (*P*<0.0001)	−0.081 (*P* = 0.19)	−0.48 (*P*<0.0001)	−0.42 (*P*<0.0001)	−0.50 (*P*<0.0001)	−0.45 (*P*<0.0001)
	Women	−0.43 (*P*<0.0001)	−0.37 (*P*<0.0001)	−0.044 (*P* = 0.42)	−0.45 (*P*<0.0001)	−0.34 (*P*<0.0001)	−0.45 (*P*<0.0001)	−0.46 (*P*<0.0001)
MCRI	All	−0.24 (*P*<0.0001)	−0.22 (*P*<0.0001)	−0.045 (*P* = 0.27)	−0.22 (*P*<0.0001)	−0.15 (*P* = 0.0002)	−0.22 (*P*<0.0001)	−0.15 (*P* = 0.0005)
	Men	−0.28 (*P*<0.0001)	−0.27 (*P*<0.0001)	−0.004 (*P* = 0.95)	−0.21 (*P* = 0.0007)	−0.27 (*P*<0.0001)	−0.23 (*P* = 0.0002)	−0.24 (*P* = 0.0002)
	Women	−0.23 (*P*<0.0001)	−0.19 (*P* = 0.0005)	−0.15 (*P* = 0.0078)	−0.26 (*P*<0.0001)	−0.12 (*P* = 0.033)	−0.22 (*P*<0.0001)	−0.21 (*P* = 0.0002)
Fasting Glucose	All	0.24 (*P*<0.0001)	0.15 (*P*<0.0001)	0.086 (*P* = 0.024)	0.20 (*P*<0.0001)	0.064 (*P* = 0.093)	0.17 (*P*<0.0001)	0.007 (*P* = 0.87)
	Men	0.11 (*P* = 0.054)	0.074 (*P* = 0.20)	−0.016 (*P* = 0.78)	0.091 (*P* = 0.12)	0.084 (*P* = 0.15)	0.11 (*P* = 0.062)	0.12 (*P* = 0.0496)
	Women	0.30 (*P*<0.0001)	0.23 (*P*<0.0001)	0.024 (*P* = 0.64)	0.22 (*P*<0.0001)	0.20 (*P*<0.0001)	0.22 (*P*<0.0001)	0.13 (*P* = 0.015)
2-hour Glucose	All	0.17 (*P*<0.0001)	0.16 (*P*<0.0001)	−0.19 (*P*<0.0001)	0.12 (*P* = 0.0014)	0.25 (*P*<0.0001)	0.26 (*P*<0.0001)	0.26 (*P*<0.0001)
	Men	0.19 (*P* = 0.0013)	0.098 (*P* = 0.094)	−0.11 (*P* = 0.052)	0.18 (*P* = 0.002)	0.17 (*P* = 0.0033)	0.26 (*P*<0.0001)	0.21 (*P* = 0.0007)
	Women	0.23 (*P*<0.0001)	0.18 (*P* = 0.0004)	−0.13 (*P* = 0.0099)	0.21 (*P*<0.0001)	0.23 (*P*<0.0001)	0.27 (*P*<0.0001)	0.30 (*P*<0.0001)
Fasting Insulin	All	0.54 (*P*<0.0001)	0.49 (*P*<0.0001)	0.084 (*P* = 0.038)	0.52 (*P*<0.0001)	0.35 (*P*<0.0001)	0.55 (*P*<0.0001)	0.30 (*P*<0.0001)
	Men	0.60 (*P*<0.0001)	0.52 (*P*<0.0001)	0.087 (*P* = 0.15)	0.57 (*P*<0.0001)	0.46 (*P*<0.0001)	0.60 (*P*<0.0001)	0.49 (*P*<0.0001)
	Women	0.55 (*P*<0.0001)	0.49 (*P*<0.0001)	0.20 (*P* = 0.0003)	0.57 (*P*<0.0001)	0.38 (*P*<0.0001)	0.52 (*P*<0.0001)	0.40 (*P*<0.0001)
Carotid IMT	All	0.17 (*P*<0.0001)	0.095 (*P* = 0.016)	0.06 (*P* = 0.13)	0.18 (*P*<0.0001)	0.035 (*P* = 0.38)	0.17 (*P*<0.0001)	−0.014 (*P* = 0.73)
	Men	0.11 (*P* = 0.069)	−0.016 (*P* = 0.80)	−0.033 (*P* = 0.59)	0.039 (*P* = 0.53)	0.008 (*P* = 0.90)	0.058 (*P* = 0.34)	−0.025 (*P* = 0.69)
	Women	0.18 (*P* = 0.0004)	0.18 (*P* = 0.0004)	−0.016 (*P* = 0.76)	0.23 (*P*<0.0001)	0.18 (*P* = 0.0005)	0.25 (*P*<0.0001)	0.21 (*P*<0.0001)
SBP	All	0.29 (*P*<0.0001)	0.17 (*P*<0.0001)	0.22 (*P*<0.0001)	0.33 (*P*<0.0001)	−0.002 (*P* = 0.95)	0.25 (*P*<0.0001)	−0.038 (*P* = 0.35)
	Men	0.31 (*P*<0.0001)	0.22 (*P*<0.0001)	0.11 (*P* = 0.057)	0.31 (*P*<0.0001)	0.16 (*P* = 0.0073)	0.30 (*P*<0.0001)	0.24 (*P*<0.0001)
	Women	0.23 (*P*<0.0001)	0.19 (*P* = 0.0002)	0.04 (*P* = 0.43)	0.25 (*P*<0.0001)	0.17 (*P* = 0.0008)	0.25 (*P*<0.0001)	0.26 (*P*<0.0001)
DBP	All	0.18 (*P*<0.0001)	0.029 (*P* = 0.44)	0.19 (*P*<0.0001)	0.20 (*P*<0.0001)	−0.093 (*P* = 0.014)	0.12 (*P* = 0.0022)	−0.066 (*P* = 0.10)
	Men	0.18 (*P* = 0.0018)	0.025 (*P* = 0.67)	0.059 (*P* = 0.31)	0.14 (*P* = 0.015)	−0.009 (*P* = 0.88)	0.13 (*P* = 0.025)	0.18 (*P* = 0.0025)
	Women	0.12 (*P* = 0.019)	0.074 (*P* = 0.14)	0.026 (*P* = 0.60)	0.12 (*P* = 0.016)	0.07 (*P* = 0.16)	0.12 (*P* = 0.02)	0.19 (*P* = 0.0003)
CRP	All	0.36 (*P*<0.0001)	0.42 (*P*<0.0001)	−0.19 (*P* = 0.0003)	0.30 (*P*<0.0001)	0.44 (*P*<0.0001)	0.46 (*P*<0.0001)	0.51 (*P*<0.0001)
	Men	0.28 (*P* = 0.0004)	0.22 (*P* = 0.0071)	0.02 (*P* = 0.81)	0.27 (*P* = 0.0006)	0.20 (*P* = 0.013)	0.29 (*P* = 0.0002)	0.33 (*P*<0.0001)
	Women	0.54 (*P*<0.0001)	0.50 (*P*<0.0001)	0.042 (*P* = 0.55)	0.57 (*P*<0.0001)	0.45 (*P*<0.0001)	0.58 (*P*<0.0001)	0.60 (*P*<0.0001)
Adiponectin	All	−0.28 (*P*<0.0001)	−0.14 (*P* = 0.0064)	−0.16 (*P* = 0.0017)	−0.29 (*P*<0.0001)	−0.011 (*P* = 0.83)	−0.23 (*P*<0.0001)	0.079 (*P* = 0.14)
	Men	−0.19 (*P* = 0.016)	−0.15 (*P* = 0.069)	0.089 (*P* = 0.27)	−0.18 (*P* = 0.027)	−0.21 (*P* = 0.0093)	−0.23 (*P* = 0.0033)	−0.14 (*P* = 0.090)
	Women	−0.28 (*P*<0.0001)	−0.20 (*P* = 0.0041)	−0.12 (*P* = 0.079)	−0.29 (*P*<0.0001)	−0.12 (*P* = 0.072)	−0.25 (*P* = 0.0002)	−0.11 (*P* = 0.11)
PAI-1	All	0.34 (*P*<0.0001)	0.28 (*P*<0.0001)	0.008 (*P* = 0.88)	0.33 (*P*<0.0001)	0.21 (*P*<0.0001)	0.37 (*P*<0.0001)	0.15 (*P* = 0.0059)
	Men	0.33 (*P*<0.0001)	0.24 (*P* = 0.0027)	−0.024 (*P* = 0.76)	0.36 (*P*<0.0001)	0.25 (*P* = 0.0015)	0.40 (*P*<0.0001)	0.36 (*P*<0.0001)
	Women	0.33 (*P*<0.0001)	0.33 (*P*<0.0001)	−0.066 (*P* = 0.34)	0.31 (*P*<0.0001)	0.34 (*P*<0.0001)	0.35 (*P*<0.0001)	0.27 (*P* = 0.0001)

Data are correlation coefficients with *P* values in parentheses.

### Comparison of the Correlations of BAI and BMI with Cardiometabolic Risk Factors

Comparison of BAI and BMI in the strength of their correlations with cardiometabolic traits (Table 3) revealed the following. BMI was more strongly correlated than BAI with TG, M/I, fasting insulin and SBP in all analyses (both sex-pooled and sex-stratified). BMI was also more strongly correlated than BAI with LDL-C, HDL-C, MCRI, fasting glucose, carotid IMT, DBP, adiponectin, and PAI-1 in sex-pooled analyses; with HDL-C, 2-hour glucose, DBP, CRP, and PAI-1 in men; and with MCRI, carotid IMT, CRP, and adiponectin in women. BMI and BAI were similar in the strength of their correlations with 2-hour glucose and CRP in sex-pooled data; with MCRI, fasting glucose, LDL-C, adiponectin, carotid IMT in men; and with LDL-C, HDL-C, 2-hour glucose, fasting glucose, DBP, and PAI-1 in women.

**Table 3:** Comparison of correlation coefficients between cardiometabolic risk factors and BMI versus BAI.

Variable	BMI correlation coefficient	BAI correlation coefficient	*P* value^a^
**SEX-POOLED**			
PBF	0.51	**0.78**	<0.0001
LDL-C	**0.14**	0.033	0.0006
HDL-C	−**0.27**	−0.008	<0.0001
TG	**0.32**	0.054	<0.0001
M/I	−**0.46**	−0.37	0.001
MCRI	−**0.22**	−0.15	0.019
Fasting Glucose	**0.17**	0.064	0.0006
2-hour Glucose	0.26	0.25	0.72
Fasting Insulin	**0.55**	0.35	<0.0001
Carotid IMT	**0.17**	0.035	<0.0001
SBP	**0.25**	−0.002	<0.0001
DBP	**0.12**	−0.093	<0.0001
CRP	0.46	0.44	0.52
Adiponectin	−**0.23**	−0.011	<0.0001
PAI-1	**0.37**	0.21	<0.0001
**MEN**			
PBF	**0.79**	0.63	<0.0001
LDL-C	0.14	0.15	0.82
HDL-C	−**0.32**	−0.21	0.0066
TG	**0.36**	0.23	0.0012
M/I	−**0.50**	−0.42	0.038
MCRI	−0.23	−0.27	0.42
Fasting Glucose	0.11	0.084	0.58
2-hour glucose	**0.26**	0.17	0.046
Fasting Insulin	**0.6**	0.46	<0.0001
Carotid IMT	0.058	0.008	0.25
SBP	**0.3**	0.16	0.0008
DBP	**0.13**	−0.009	0.0012
CRP	**0.29**	0.2	0.029
Adiponectin	−0.23	−0.21	0.54
PAI-1	**0.4**	0.25	0.0002
**WOMEN**			
PBF	**0.77**	0.69	<0.0001
LDL-C	0.16	0.12	0.27
HDL-C	−0.27	−0.21	0.054
TG	**0.33**	0.24	0.0078
M/I	−**0.45**	−0.34	0.0004
MCRI	−**0.22**	−0.12	0.0014
Fasting Glucose	0.22	0.2	0.53
2-hour glucose	0.27	0.23	0.23
Fasting Insulin	**0.52**	0.38	<0.0001
Carotid IMT	**0.25**	0.18	0.042
SBP	**0.25**	0.17	0.011
DBP	0.12	0.07	0.16
CRP	**0.58**	0.45	<0.0001
Adiponectin	−**0.25**	−0.12	<0.0001
PAI-1	0.35	0.34	0.67

Correlation coefficients that are significantly greater are highlighted in bold.

a *P* values from Hotelling’s T-test.

### Comparison of the Correlations of Hip Circumference and Weight with Cardiometabolic Risk Factors

We compared the two variables that distinguish the calculations of BAI and BMI, namely hip circumference and weight, respectively (Table S2). Weight was more strongly correlated than hip circumference with TG and SBP in all analyses (both sex-pooled and sex-stratified); in sex-pooled data with LDL-C, HDL-C, carotid IMT, DBP, and adiponectin; in men with 2-hour glucose, DBP, and PAI-1; and in women with M/I, MCRI, fasting insulin, CRP and adiponectin. Hip circumference outperformed weight in the strength of its association with only CRP in pooled data. Hip circumference and weight were similarly associated with all other cardiometabolic traits.

### Comparison of the Correlations of Anthropometric Variables with PBF

**Figure 1:**
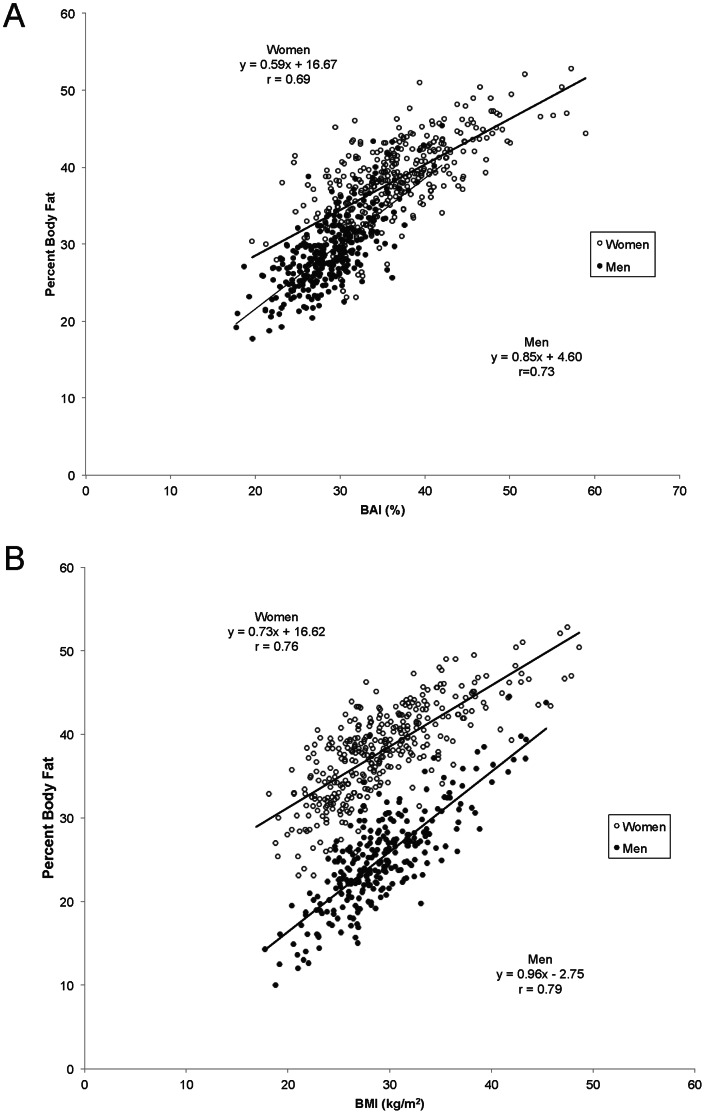
Relationship of percent body fat (PBF) with body adiposity index (BAI) and body mass index (BMI) in men and women. A. BAI versus PBF. B. BMI versus PBF. The graphs are generated on untransformed data for the BAI and BMI variables, while the main analyses in our study are based on log transformations of these variables. Therefore, the correlation coefficients (r) here are slightly different than those reported elsewhere in the text.

Comparison of the various anthropometric variables in their ability to predict PBF is displayed in Table 2. BAI outperformed BMI in the strength of its correlation with DXA-derived PBF in sex-pooled analysis; however, when data was sex-stratified, BAI was weaker than BMI in predicting PBF in men and women. Figure 1 illustrates these findings. The larger percentage of overlapping data points between men and women when PBF is plotted against BAI leads to greater correlation between BAI and PBF than between BMI and PBF (r = 0.78 versus r = 0.51; *P*<0.0001) in sex-pooled analyses. However, in sex-stratified data, correlations with PBF were greater for BMI than for BAI (in men, r = 0.79 versus r = 0.63, *P*<0.0001; in women, r = 0.77 versus r = 0.69, *P*<0.0001) (Figure 1). Similarly, when DXA-derived total fat mass was examined, both BMI and BAI correlated with this measure. However, BMI was stronger than BAI in its correlation with total fat mass in both sex-pooled (r = 0.84 versus r = 0.71; P<0.0001) and sex-stratified analyses (in men, r = 0.88 versus r = 0.61, P<0.0001; in women, r = 0.91 versus r = 0.71, P<0.0001).

After BAI, height was the second strongest correlate with PBF in sex-pooled data (r = −0.54). However, height lost its correlation with PBF in sex-stratified analyses (Figure S1); conversely, waist, hip, weight, and BMI all strengthened in their correlations with PBF in sex-stratified analyses.

## Discussion

We found that the cardiometabolic disease risk factors were more consistently correlated with BMI, waist circumference, and weight than with BAI. In cases wherein significant correlations were found, BAI was either similar to, or weaker than, BMI in the strength of these associations. Thus, our results show that with regards to its utility as a correlate of the cardiometabolic risk factors measured in our study, BAI is similar or inferior to the currently widely used BMI.

Our study is one of a growing number that have assessed the strength of BAI’s association with cardiometabolic risk factors [9], [13], [14], [15], [16], [17], [18], [29]. We included three variables that have not yet been studied with regards to their correlation with BAI, namely, carotid IMT, plasminogen activator inhibitor-1, and metabolic clearance rate of insulin from the euglycemic clamp. Only three other studies besides ours utilized a statistical test to compare correlation coefficients between anthropometric measures of body adiposity and cardiometabolic risk factors [9], [17], [18]. One found that waist circumference and BMI were more strongly correlated than BAI with OGTT-derived insulin sensitivity and type 2 diabetes risk [9]. The second found that BMI and waist circumference were superior to BAI in the strength of their correlations with most of the seven cardiovascular risk variables studied (LDL-C, HDL-C, TG, insulin, fasting glucose, SBP, and DBP); this superiority of BMI and waist circumference was upheld also when data was stratified by sex and age [17]. There was no case in which BAI was found to be superior to BMI or waist with regards to its correlative strength with cardiovascular risk variables [17]. Finally, the third of these studies found that while BAI was superior to BMI in its correlation with measures of leptin, BMI was more strongly correlated than BAI with adiponectin levels, HDL-C, TG, and glucose homeostasis traits (insulin, fasting glucose, and homeostasis model assessment of insulin resistance) [18].

Additional studies compared correlations of BAI and BMI with cardiometabolic traits, without formal statistical comparison of the correlation coefficients. One reported that regardless of glycemic status (euglycemic, impaired fasting glucose, impaired glucose tolerance, or type 2 diabetes mellitus), BAI had the weakest correlation with fasting glucose, 2-hour glucose, SBP, and DBP, as compared with other indices of adiposity (waist circumference, waist-to-hip ratio, waist-to-height ratio, and BMI) [14]. A study of obese post-menopausal women examined BAI’s ability to detect changes in cardiometabolic risk factors after a weight loss intervention [13]. The study found that the percent change in BAI following weight loss was significantly associated with percent changes in CRP and leptin, but not with percent change in any of the other cardiometabolic risk factors examined (total cholesterol, HDL-C, TG, fasting glucose, clamp-derived insulin sensitivity, SBP, or DBP) [13]. A study examining correlation coefficients between several anthropometric measures and metabolic risk factors (HDL-C, LDL-C, TG, fasting glucose, SBP, and DBP) concluded that BMI, waist circumference, waist-to-height ratio, and waist-to-hip ratio were all better correlated than BAI with the risk factors measured [16]. In a biracial cohort, anthropometric measures, including BAI, were found to be similarly correlated with most cardiovascular risk factors; however, BAI lacked correlation with LDL-C and total cholesterol in African-American women, and with SBP and DBP in African American men [15]. Finally, in a cohort of obese individuals classified as insulin resistant (homeostasis model assessment of insulin resistance (HOMA-IR) >2.5) and insulin sensitive, BAI and BMI differed in their correlations with cardiometabolic risk factors [29]. In each individual group, as well as combined, BAI was correlated with serum adiponectin, leptin, and CRP levels; however, no correlation was found between BAI and insulin levels or HOMA-IR. BMI, on the other hand, was not correlated with adiponectin levels in any group, but was correlated with leptin, CRP, insulin, and HOMA-IR in the insulin-resistant group [29].

In our study, BAI was significantly associated with several of the cardiometabolic risk factors, and in several cases performed similarly to BMI in the strength of its correlations with these risk factors (Table 3). Within the four categories examined (glucose homeostasis traits, lipid parameters, cardiovascular traits, and biomarkers), there was no clear pattern of association, or lack thereof, with respect to BAI. Further, with certain variables studied (e.g. LDL-C, TG, fasting glucose, carotid IMT, SBP, and adiponectin), no correlation was found with either BAI or PBF when analyzing sex-pooled data; in most cases, correlations of BAI and PBF with these traits were only revealed when the data was sex-stratified. This may be explained by differences between men and women with respect to these variables, as is demonstrated in the clinical characteristics of our cohort (Table 1). Nevertheless, comparisons of BMI and BAI with respect to their correlations with these variables within sex-stratified data showed that BMI was superior to BAI in its associations with SBP and TG in both men and women, as well as adiponectin and carotid IMT in women. BMI and BAI were similar in their associations with LDL-C and fasting glucose in sex-stratified analyses. Therefore, variation between the data in men and women with respect to BAI is unlikely to fully account for the relative weakness of its associations with cardiometabolic risk factors.

To understand the differences between BAI and BMI in the strength of their correlations with cardiometabolic risk factors, we compared hip and weight, the two variables that differ in the calculations of these adiposity indices. We found that weight was more associated with many of the cardiometabolic risk factors examined, consistent with the findings comparing BAI and BMI.

The results of our study illustrate the complexity of the task of identifying accurate markers of cardiometabolic risk factors associated with obesity. We found that PBF was less consistently correlated with several of the cardiometabolic risk factors than BMI. This suggests that BMI’s relationship with total adiposity may not be the sole determinate of its relationship with cardiometabolic risk. Further research is needed to clarify the characteristics of anthropometric variables that determine their associations with cardiometabolic risk factors and disease.

We also aimed to identify a single anthropometric measurement or index that most accurately predicted body adiposity as measured by DXA. While BAI did correlate most strongly with PBF in sex-pooled data analyses, BMI proved the most accurate tool to predict PBF in men and women in sex-stratified analyses, a finding supported in a recent study performed on a large German cohort [9]. These findings indicate that BMI is better able to account for the differences in body fat content and distribution in men and women, a conclusion that conflicts with recent data in a cohort of women with familial lipodystrophy, which suggested that BAI may be a more sensitive mode of estimating adiposity [11]. Our findings suggest that sex-adjustment of current BMI cutoffs for defining normal weight, overweight, and obese, might yield a more accurate assessment of body adiposity than either BMI or BAI as currently used.

BAI was designed specifically as a single equation that could predict body adiposity in pooled analyses of both genders. It was not designed as a tool to predict cardiometabolic risk. Our current data verifies that BAI is a stronger correlate of PBF than BMI in sex-pooled data, providing further support that BAI achieves what it was designed to do. Recent literature has raised questions about sex-specific bias in the variables used to derive BAI [9]. We found that among the characteristics considered for inclusion in the BAI equation (i.e. waist, hip, height, and weight), hip and height did, in fact, show the strongest correlation with PBF in sex-pooled data, in agreement with Bergman *et al*. [7]. However, as described in recent articles and the results herein, the correlation between height and adiposity is lost in sex-stratified analysis, suggesting that the correlation between height and PBF may be driven by sex-differences in these variables [9], [19] (Figure S1). However, the significance of this finding with respect to the comparative analysis of BAI and BMI is unclear, given that both indices incorporate height into their calculations.

We acknowledge limitations in our study, including the lack of racial and ethnic diversity in our cohort. We studied Mexican Americans, as did Bergman *et al*. [7], which provided us with the ability to test BAI’s performance in a population representative of its derivation group. Nevertheless, the results herein may not apply to other ethnic groups. Additionally, our study did not have access to longitudinal follow-up data on study subjects, which would have provided us with cardiovascular and metabolic disease outcomes, such as incident myocardial infarction and diabetes. Further studies are needed to examine BAI as it relates to disease outcomes, and to clarify any other clinical role that BAI may play in the evaluation of obesity. At the present time, if the goal is assessment of cardiometabolic risk, BMI is a more suitable tool than BAI.

## Supporting Information

Figure S1 **Relationship between height and percent body fat (PBF) in sex-pooled (A) and sex-stratified (B) analyses.**
(TIFF)

Table S1 **Correlations of anthropometric measurements with cardiometabolic risk factors taking family relationships into consideration**
(DOCX)

Table S2 **Comparison of correlation coefficients between cardiometabolic risk factors and hip circumference versus weight.**
(DOCX)
