# Supplementary figures and images for: Body Adiposity Index versus Body Mass Index and Other Anthropometric Traits as Correlates of Cardiometabolic Risk Factors
Source: PLoS One. 2013 Jun 11;8(6):e65954. doi: 10.1371/journal.pone.0065954 (PMC3679008; doi:10.1371/journal.pone.0065954)

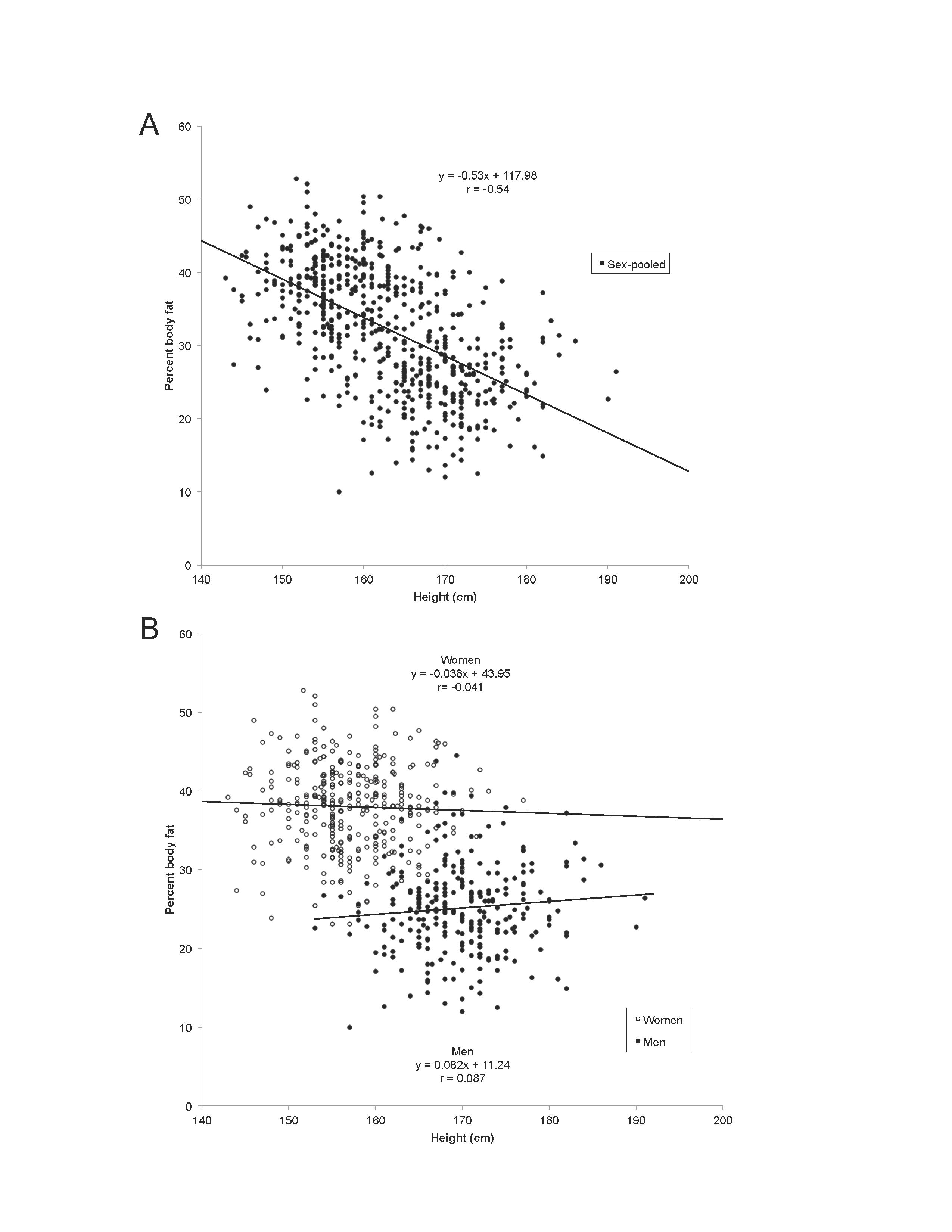

Supplement: Figure S1 — Relationship between height and percent body fat (PBF) in sex-pooled (A) and sex-stratified (B) analyses. (TIFF) [file pone.0065954.s001.tiff]
