# Supplementary material for: Body Adiposity Index versus Body Mass Index and Other Anthropometric Traits as Correlates of Cardiometabolic Risk Factors
Source: PLoS One. 2013 Jun 11;8(6):e65954. doi: 10.1371/journal.pone.0065954 (PMC3679008; doi:10.1371/journal.pone.0065954)
Supplement: Table S1 — Correlations of anthropometric measurements with cardiometabolic risk factors taking family relationships into consideration (DOCX) [file pone.0065954.s002.docx]

Table S1. Correlations of anthropometric measurements with cardiometabolic risk factors taking family relationships into consideration

|  |  | Waist | Hip | Height | Weight | BAI | BMI | PBF |
| --- | --- | --- | --- | --- | --- | --- | --- | --- |
| PBF (DXA) | All | 0.28 (*P*<0.0001) | 0.54 (*P*<0.0001) | -0.55 (*P*<0.0001) | 0.15 (*P*<0.0001) | 0.80 (*P*<0.0001) | 0.51 (*P*<0.0001) |  |
|  | Men | 0.77 (*P*<0.0001) | 0.71 (*P*<0.0001) | 0.10 (*P*=0.097) | 0.76 (*P*<0.0001) | 0.63 (*P*<0.0001) | 0.79 (*P*<0.0001) |  |
|  | Women | 0.68 (*P*<0.0001) | 0.69 (*P*<0.0001) | -0.047 (*P*=0.33) | 0.72 (*P*<0.0001) | 0.70 (*P*<0.0001) | 0.77 (*P*<0.0001) |  |
| LDL-C | All | 0.19 (*P*<0.0001) | 0.060 (*P*=0.093) | 0.048 (*P*=0.18) | 0.15 (*P*<0.0001) | 0.024 (*P*=0.52) | 0.14 (*P*<0.0001) | -0.013 (*P*=0.76) |
|  | Men | 0.14 (*P*=0.023) | 0.085 (*P*=0.13) | -0.071 (*P*=0.22) | 0.10 (*P*=0.11) | 0.15 (*P*=0.0067) | 0.14 (*P*=0.022) | 0.081 (*P*=0.26) |
|  | Women | 0.17 (*P*=0.00026) | 0.079 (*P*=0.077) | -0.091 (*P*=0.037) | 0.11 (*P*=0.027) | 0.12 (*P*=0.0053) | 0.15 (*P*=0.00059) | 0.18 (*P*=0.00024) |
| HDL-C | All | -0.33 (*P*<0.0001) | -0.22 (*P*<0.0001) | -0.27 (*P*<0.0001) | -0.39 (*P*<0.0001) | -0.0086 (*P*=0.84) | -0.29 (*P*<0.0001) | 0.11 (*P*=0.016) |
|  | Men | -0.31 (*P*<0.0001) | -0.27 (*P*<0.0001) | -0.11 (*P*=0.06) | -0.35 (*P*<0.0001) | -0.21 (*P*=0.00017) | -0.34 (*P*<0.0001) | -0.27 (*P*<0.0001) |
|  | Women | -0.27 (*P*<0.0001) | -0.26 (*P*<0.0001) | -0.095 (*P*=0.02) | -0.29 (*P*<0.0001) | -0.21 (*P*=0.00014) | -0.28 (*P*<0.0001) | -0.14 (*P*=0.015) |
| TG | All | 0.35 (*P*<0.0001) | 0.17 (*P*<0.0001) | 0.18 (*P*<0.0001) | 0.36 (*P*<0.0001) | 0.030 (*P*=0.43) | 0.31 (*P*<0.0001) | -0.041 (*P*=0.37) |
|  | Men | 0.36 (*P*<0.0001) | 0.24 (*P*=0.00012) | 0.041 (*P*=0.48) | 0.35 (*P*<0.0001) | 0.23 (*P*<0.0001) | 0.37 (*P*<0.0001) | 0.29 (*P*=0.00017) |
|  | Women | 0.31 (*P*<0.0001) | 0.21 (*P*<0.0001) | -0.074 (*P*=0.15) | 0.27 (*P*<0.0001) | 0.24 (*P*<0.0001) | 0.32 (*P*<0.0001) | 0.21 (*P*<0.0001) |
| M/I | All | -0.42 (*P*<0.0001) | -0.40 (*P*<0.0001) | 0.053 (*P*=0.22) | -0.36 (*P*<0.0001) | -0.37 (*P*<0.0001) | -0.46 (*P*<0.0001) | -0.36 (*P*<0.0001) |
|  | Men | -0.53 (*P*<0.0001) | -0.47 (*P*<0.0001) | -0.072 (*P*=0.26) | -0.48 (*P*<0.0001) | -0.43 (*P*<0.0001) | -0.51 (*P*<0.0001) | -0.47 (*P*<0.0001) |
|  | Women | -0.43 (*P*<0.0001) | -0.36 (*P*<0.0001) | -0.033 (*P*=0.54) | -0.44 (*P*<0.0001) | -0.35 (*P*<0.0001) | -0.45 (*P*<0.0001) | -0.46 (*P*<0.0001) |
| MCRI | All | -0.25 (*P*<0.0001) | -0.22 (*P*<0.0001) | -0.029 (*P*=0.49) | -0.22 (*P*<0.0001) | -0.16 (*P*<0.0001) | -0.25 (*P*<0.0001) | -0.17 (*P*<0.0001) |
|  | Men | -0.30 (*P*<0.0001) | -0.28 (*P*<0.0001) | -0.0049 (*P*=0.94) | -0.24 (*P*<0.0001) | -0.28 (*P*<0.0001) | -0.27 (*P*<0.0001) | -0.26 (*P*<0.0001) |
|  | Women | -0.20 (*P*=0.00043) | -0.16 (*P*=0.0042) | -0.13 (*P*=0.0093) | -0.24 (*P*<0.0001) | -0.10 (*P*=0.057) | -0.20 (*P*=0.00016) | -0.21 (*P*<0.0001) |
| Fasting Glucose | All | 0.22 (*P*<0.0001) | 0.14 (*P*=0.0072) | 0.11 (*P*=0.0049) | 0.18 (*P*=0.00015) | 0.026 (*P*=0.48) | 0.14 (*P*=0.0009) | -0.030 (*P*=0.48) |
|  | Men | 0.050 (*P*=0.37) | 0.0045 (*P*=0.91) | -0.050 (*P*=0.29) | 0.0073 (*P*= 0.88) | 0.035 (*P*=0.38) | 0.031 (*P*=0.57) | 0.10 (*P*=0.032) |
|  | Women | 0.29 (*P*<0.0001) | 0.24 (*P*=0.0028) | 0.040 (*P*=0.26) | 0.21 (*P*=0.0096) | 0.20 (*P*=0.0049) | 0.21 (*P*=0.012) | 0.11 (*P*=0.042) |
| 2-hour Glucose | All | 0.17 (*P*<0.0001) | 0.16 (*P*<0.0001) | -0.18 (*P*<0.0001) | 0.12 (*P*=0.0016) | 0.25 (*P*<0.0001) | 0.26 (*P*<0.0001) | 0.26 (*P*<0.0001) |
|  | Men | 0.19 (*P*=0.00085) | 0.10 (*P*=0.080) | -0.11 (*P*=0.043) | 0.18 (*P*=0.00075) | 0.18 (*P*=0.0025) | 0.26 (*P*<0.0001) | 0.21 (*P*<0.0001) |
|  | Women | 0.23 (*P*<0.0001) | 0.17 (*P*=0.0005) | -0.13 (*P*=0.0059) | 0.20 (*P*<0.0001) | 0.23 (*P*<0.0001) | 0.27 (*P*<0.0001) | 0.29 (*P*<0.0001) |
| Fasting Insulin | All | 0.54 (*P*<0.0001) | 0.49 (*P*<0.0001) | 0.081 (*P*=0.062) | 0.51 (*P*<0.0001) | 0.35 (*P*<0.0001) | 0.55 (*P*<0.0001) | 0.29 (*P*<0.0001) |
|  | Men | 0.60 (*P*<0.0001) | 0.53 (*P*<0.0001) | 0.088 (*P*=0.20) | 0.58 (*P*<0.0001) | 0.46 (*P*<0.0001) | 0.61 (*P*<0.0001) | 0.51 (*P*<0.0001) |
|  | Women | 0.54 (*P*<0.0001) | 0.48 (*P*<0.0001) | 0.19 (*P*=0.00067) | 0.57 (*P*<0.0001) | 0.38 (*P*<0.0001) | 0.52 (*P*<0.0001) | 0.39 (*P*<0.0001) |
| Carotid IMT | All | 0.15 (*P*=0.0001) | 0.074 (*P*=0.040) | 0.077 (*P*=0.024) | 0.16 (*P*<0.0001) | 0.0099 (*P*=0.80) | 0.14 (*P*=0.001) | -0.053 (*P*=0.15) |
|  | Men | 0.11 (*P*=0.077) | -0.022 (*P*=0.70) | -0.034 (*P*=0.54) | 0.040 (*P*=0.54) | 0.0067 (*P*=0.90) | 0.060 (*P*=0.33) | -0.026 (*P*=0.69) |
|  | Women | 0.18 (*P*=0.00017) | 0.18 (*P*<0.0001) | -0.025 (*P*=0.66) | 0.22 (*P*<0.0001) | 0.19 (*P*=0.00015) | 0.24 (*P*<0.0001) | 0.20 (*P*<0.0001) |
| SBP | All | 0.29 (*P*<0.0001) | 0.16 (*P*<0.0001) | 0.23 (*P*<0.0001) | 0.33 (*P*<0.0001) | -0.014 (*P*=0.69) | 0.24 (*P*<0.0001) | -0.072 (*P*=0.043) |
|  | Men | 0.30 (*P*<0.0001) | 0.22 (*P*<0.0001) | 0.11 (*P*=0.041) | 0.30 (*P*<0.0001) | 0.15 (*P*=0.0017) | 0.28 (*P*<0.0001) | 0.24 (*P*<0.0001) |
|  | Women | 0.22 (*P*<0.0001) | 0.20 (*P*<0.0001) | 0.032 (*P*=0.52) | 0.24 (*P*<0.0001) | 0.18 (*P*<0.0001) | 0.24 (*P*<0.0001) | 0.24 (*P*<0.0001) |
| DBP | All | 0.19 (*P*<0.0001) | 0.030 (*P*=0.37) | 0.21 (*P*<0.0001) | 0.20 (*P*<0.0001) | -0.11 (*P*=0.0016) | 0.11 (*P*=0.0019) | -0.095 (*P*=0.012) |
|  | Men | 0.17 (*P*=0.0019) | 0.020 (*P*=0.70) | 0.060 (*P*=0.25) | 0.13 (*P*=0.037) | -0.012 (*P*=0.80) | 0.12 (*P*=0.054) | 0.17 (*P*=0.0027) |
|  | Women | 0.12 (*P*=0.012) | 0.082 (*P*=0.072) | 0.034 (*P*=0.53) | 0.12 (*P*=0.012) | 0.081 (*P*=0.093) | 0.11 (*P*=0.017) | 0.17 (*P*=0.00068) |
| CRP | All | 0.36 (*P*<0.0001) | 0.42 (*P*<0.0001) | -0.19 (*P*<0.0001) | 0.30 (*P*<0.0001) | 0.45 (*P*<0.0001) | 0.46 (*P*<0.0001) | 0.51 (*P*<0.0001) |
|  | Men | 0.29 (*P*<0.0001) | 0.22 (*P*=0.0011) | 0.025 (*P*=0.73) | 0.28 (*P*=0.00012) | 0.20 (*P*=0.0025) | 0.30 (*P*<0.0001) | 0.35 (*P*<0.0001) |
|  | Women | 0.54 (*P*<0.0001) | 0.49 (*P*<0.0001) | 0.041 (*P*=0.54) | 0.57 (*P*<0.0001) | 0.45 (*P*<0.0001) | 0.58 (*P*<0.0001) | 0.60 (*P*<0.0001) |
| Adiponectin | All | -0.28 (*P*<0.0001) | -0.15 (*P*=0.012) | -0.17 (*P*=0.00029) | -0.30 (*P*<0.0001) | -0.012 (*P*=0.82) | -0.24 (*P*<0.0001) | 0.075 (*P*=0.17) |
|  | Men | -0.19 (*P*=0.028) | -0.14 (*P*=0.12) | 0.075 (*P*=0.36) | -0.20 (*P*=0.012) | -0.21 (*P*=0.012) | -0.25 (*P*=0.00075) | -0.16 (*P*=0.045) |
|  | Women | -0.29 (*P*=0.00015) | -0.21 (*P*=0.0055) | -0.12 (*P*=0.076) | -0.31 (*P*<0.0001) | -0.14 (*P*=0.061) | -0.27 (*P*=0.00012) | -0.12 (*P*=0.087) |
| PAI-1 | All | 0.34 (*P*<0.0001) | 0.29 (*P*<0.0001) | 0.0098 (*P*=0.85) | 0.33 (*P*<0.0001) | 0.23 (*P*<0.0001) | 0.38 (*P*<0.0001) | 0.14 (*P*=0.014) |
|  | Men | 0.33 (*P*<0.0001) | 0.24 (*P*=0.00051) | -0.02 (*P*=0.77) | 0.35 (*P*<0.0001) | 0.25 (*P*=0.00038) | 0.40 (*P*<0.0001) | 0.36 (*P*<0.0001) |
|  | Women | 0.34 (*P*<0.0001) | 0.33 (*P*<0.0001) | -0.065 (*P*=0.35) | 0.31 (*P*<0.0001) | 0.35 (*P*<0.0001) | 0.36 (*P*<0.0001) | 0.27 (*P*=0.00016) |

Data are correlation coefficients with *P* values in parentheses
