# Supplementary material for: Body Adiposity Index versus Body Mass Index and Other Anthropometric Traits as Correlates of Cardiometabolic Risk Factors
Source: PLoS One. 2013 Jun 11;8(6):e65954. doi: 10.1371/journal.pone.0065954 (PMC3679008; doi:10.1371/journal.pone.0065954)
Supplement: Table S2 — Comparison of correlation coefficients between cardiometabolic risk factors and hip circumference versus weight. (DOCX) [file pone.0065954.s003.docx]

Table S2. Comparison of correlation coefficients between cardiometabolic risk factors and hip circumference versus weight

| **Variable** | **Hip correlation coefficient** | **Weight correlation coefficient** | ***P* value**^a^ |
| --- | --- | --- | --- |
| **SEX-POOLED** |  |  |  |
| PBF | **0.53** | 0.15 | <0.0001 |
| LDL-C | 0.076 | **0.15** | 0.013 |
| HDL-C | -0.22 | **-0.37** | <0.0001 |
| TG | 0.18 | **0.36** | <0.0001 |
| M/I | -0.41 | -0.37 | 0.16 |
| MCRI | -0.22 | -0.22 | 0.97 |
| Fasting Glucose | 0.15 | 0.2 | 0.13 |
| 2-hour glucose | 0.16 | 0.12 | 0.2 |
| Fasting Insulin | 0.49 | 0.52 | 0.34 |
| Carotid IMT | 0.095 | **0.18** | 0.0036 |
| SBP | 0.17 | **0.33** | <0.0001 |
| DBP | 0.029 | **0.2** | <0.0001 |
| CRP | **0.42** | 0.3 | <0.0001 |
| Adiponectin | -0.14 | **-0.29** | <0.0001 |
| PAI-1 | 0.28 | 0.33 | 0.059 |
| **MEN** |  |  |  |
| PBF | 0.7 | **0.76** | 0.017 |
| LDL-C | 0.11 | 0.1 | 0.91 |
| HDL-C | -0.26 | -0.33 | 0.076 |
| TG | 0.23 | **0.33** | 0.0042 |
| M/I | -0.47 | -0.48 | 0.93 |
| MCRI | -0.27 | -0.21 | 0.081 |
| Fasting Glucose | 0.074 | 0.091 | 0.64 |
| 2-hour glucose | 0.098 | **0.18** | 0.024 |
| Fasting Insulin | 0.52 | 0.57 | 0.084 |
| Carotid IMT | -0.016 | 0.039 | 0.13 |
| SBP | 0.22 | **0.31** | 0.014 |
| DBP | 0.025 | **0.14** | 0.0014 |
| CRP | 0.22 | 0.27 | 0.11 |
| Adiponectin | -0.15 | -0.18 | 0.39 |
| PAI-1 | 0.24 | **0.36** | 0.0006 |
| **WOMEN** |  |  |  |
| PBF | 0.68 | 0.71 | 0.13 |
| LDL-C | 0.087 | 0.11 | 0.39 |
| HDL-C | -0.26 | -0.29 | 0.28 |
| TG | 0.21 | **0.27** | 0.025 |
| M/I | -0.37 | **-0.45** | 0.0022 |
| MCRI | -0.19 | **-0.26** | 0.0076 |
| Fasting Glucose | 0.23 | 0.22 | 0.72 |
| 2-hour glucose | 0.18 | 0.21 | 0.36 |
| Fasting Insulin | 0.49 | **0.57** | 0.0008 |
| Carotid IMT | 0.18 | 0.23 | 0.12 |
| SBP | 0.19 | **0.25** | 0.037 |
| DBP | 0.074 | 0.12 | 0.1 |
| CRP | 0.5 | **0.57** | 0.0018 |
| Adiponectin | -0.20 | **-0.29** | 0.0012 |
| PAI-1 | 0.33 | 0.31 | 0.51 |

Correlation coefficients that are significantly greater are highlighted in bold

^a^*P* values from Hotelling’s T-test
